# Side effects of the metacognitive training for depression compared to a cognitive remediation training in patients with depression

**DOI:** 10.1038/s41598-021-87198-8

**Published:** 2021-04-12

**Authors:** Mona Dietrichkeit, Marion Hagemann-Goebel, Yvonne Nestoriuc, Steffen Moritz, Lena Jelinek

**Affiliations:** 1 Department of Psychiatry and Psychotherapy, University Medical Center Hamburg-Eppendorf, Hamburg, Germany; 2 Department of Psychiatry and Psychotherapy, Asklepios Clinic North, Hamburg, Germany; 3 Institute of Systems Neuroscience, University Medical Center Hamburg-Eppendorf, Hamburg, Germany; 4 Clinical Psychology, Helmut-Schmidt-University/University of the Federal Armed Forces Hamburg, Hamburg, Germany

**Keywords:** Randomized controlled trials, Depression

## Abstract

Although awareness of side effects over the course of psychotherapy is growing, side effects are still not always reported. The purpose of the present study was to examine side effects in a randomized controlled trial comparing Metacognitive Training for Depression (D-MCT) and a cognitive remediation training in patients with depression. 84 patients were randomized to receive either D-MCT or cognitive remediation training (MyBrainTraining) for 8 weeks. Side effects were assessed after the completion of each intervention (post) using the Short Inventory of the Assessment of Negative Effects (SIAN) and again 6 months later (follow-up) using the Negative Effects Questionnaire (NEQ). D-MCT and MyBrainTraining did not differ significantly in the number of side effects. At post assessment, 50% of the D-MCT group and 59% of the MyBrainTraining group reported at least one side effect in the SIAN. The most frequently reported side effect was disappointment in subjective benefit of study treatment. At follow-up, 52% reported at least one side effect related to MyBrainTraining, while 34% reported at least one side effect related to the D-MCT in the NEQ. The most frequently reported side effects fell into the categories of “symptoms” and “quality”. Our NEQ version was missing one item due to a technical error. Also, allegiance effects should be considered. The sample size resulted in low statistical power. The relatively tolerable number of side effects suggests D-MCT and MyBrainTraining are safe and well-received treatment options for people with depression. Future studies should also measure negative effects to corroborate our results.

## Introduction

Scott and Young have postulated that “every branch of medicine learns from mistakes, the same must surely be true for psychological treatments” (p. 208)^1^. Reviews suggest that pharmacotherapy research still pays greater attention to negative treatment effects than psychotherapy research does^2–4^. However, it is often difficult to classify what counts as a negative or adverse event in psychotherapy^5^, which might be one reason for the lack of attention to this issue in psychotherapy studies. A number of terms and considerations have been suggested^5–7^. All negative or unwanted occurrences during or following treatment, irrespective of the supposed cause, should be termed *unwanted events*. If the therapy is the supposed cause of unwanted events, *adverse treatment reactions* or *side effects* caused by correctly applied treatment can be distinguished from *malpractice* and *unethical behavior* caused by incorrectly applied treatment. Together, side effects, malpractice, and unethical behavior are *negative effects* (i.e., effects of treatment). Based on Linden and colleagues^7^, it is important to note that *side effects* can only occur if the treatment is correctly applied. Side effects cannot occur in the event of malpractice or unethical conduct as they are the products only of a correctly applied treatment (*lege artis*). *Treatment non-response* or *deterioration of illness* can also be counted as an unwanted event, as well as the manifestation of *novel symptoms.* Finally, *dropout* should be considered as an unwanted event^8,9^. However, possible reasons for dropouts are many and should thus be considered in line with O’Keeffe et al.^9^, who argue that a dropout represents an unwanted event if patients drop out, for example, because they dislike the treatment or feel they have not benefited from it (“dissatisfied” dropout^9^). Yet, a dropout might also be due to symptom improvement^10,11^ (“got-what-they-needed” dropout^9^) or practical reasons (e.g., time constraints, transportation)^12^ and is thus not necessarily an unwanted event.

Although there is growing awareness of unwanted events and side effects in psychotherapy, the current evidence is still sparse^4^, and definitions, terminology, and numbers tend to diverge. In a cross-sectional survey of 14,587 people receiving psychological treatment from 184 different services in England and Wales, 5.2% of the patients reported lasting unwanted effects and, of those patients receiving cognitive behavioral therapy (CBT), 4% reported lasting negative effects^13^. In an online sample of 195 former psychotherapy patients in Germany suffering from various disorders, 93.8% reported at least one negative effect measured with the Inventory for the Assessment of Negative Effects of Psychotherapy (INEP) and the highest rates of negative effects were reported for intrapersonal changes (15.8%), stigmatization (14.9%), and relationships (12%)^14^. Another online sample of 135 patients with depression used the Positive and Negative Effects of Psychotherapy Scale (PANEPS) and found that 52.6% of participants reported at least one unwanted event, with side effects (e.g., fear of stigmatization) and malpractice (e.g., verbal abuse/mockery, therapist being absent-minded) more prevalent compared to unethical conduct (e.g., intolerance, sexual harassment)^15^. Deterioration of mood state and/or unwanted treatment reactions as measured with the Unwanted Events and Adverse Treatment Reactions (UE-G)^16^ were experienced by 60–65% of all patients suffering from various mental disorders in an inpatient group psychotherapy^8^. In another study using the INEP^14^, 58.7% of a patient group from a psychiatric hospital and 45.2% of a patient group from a psychosomatic hospital reported at least one negative effect^17^. A German study on outpatient CBT using the INEP^14^ reported that 84% of a sample with various mental disorders reported at least one side effect, most commonly symptom-specific changes (e.g., feeling worse)^18^. A different more recent study^19^ using the INEP on inpatients and outpatients reported lower numbers: At least one side effect was reported by 37.3% of inpatients and 15.2% of outpatients^19^.

Furthermore, the following risk factors for side effects have been identified: poor therapeutic allegiance^14,18^, current interpersonal difficulties^19^, unfulfilled expectations regarding therapy outcome, inpatient treatment compared to outpatient treatment^14,19^, a number of pretreatment^18^ or prior experience with psychotherapy^19^, older age^15^, higher motivation for psychotherapy^19^, less optimism toward the treatment, and treatment in a psychiatric compared to a psychosomatic hospital^17^. Furthermore, there is tentative evidence for differences between different therapeutic approaches: CBT patients felt more pressured to perform interventions compared to other therapeutic approaches, while patients receiving psychodynamic therapy felt offended more often by something their therapist said^14^.

To our knowledge, side effects of cognitive remediation training (CRT) have hardly been investigated. Rose et al. concluded from their study on satisfaction and side effects of CRT that the lack of subjective improvement during the training may have a detrimental effect on self-esteem^20^.

Accumulating evidence supports the common notion that no treatment is without side effects irrespective of the disorder. Thus, side effects occur quite regularly in psychotherapy and researchers are advocating the reporting of side effects that occur during or following the intervention being studied^3,4^.

In this study, we focus on side effects of the Metacognitive Training for Depression (D-MCT). The D-MCT^21^ was derived from the Metacognitive Training for Psychosis^22^ and aims to challenge cognitive as well as metacognitive biases via insight-based corrective experiences that are provided through playful exercises. It shows some overlap with CBT^23^ but is considered a “low-threshold” group treatment both by therapists as well as patients. Because of its high standardization (it is administered with the help of a slide-based presentation), it is easy to administer, and even hesitant or skeptical patients may benefit as they are not required to engage in discussions or share personal examples during the sessions. Current research on the D-MCT supports its positive impact on depressive symptoms and depression-specific cognitive biases^24–28^. The D-MCT has been shown to be superior in the reduction of depressive symptoms compared to an active control intervention immediately after the intervention and at 6-month follow-up^25^ as well as after 3.5 years^26^. However, additional RCT studies, especially from independent research groups, are needed to fully assess its efficacy. Also, the D-MCT showed high acceptance among patients in two studies^29^ in which the majority of patients (85–94%) reported that they would recommend the D-MCT to others and considered it “useful and sensible” (83–100%). Although the D-MCT seems to be well accepted among patients, until now its side effects have not been evaluated. To close this research gap, the present study aimed to identify the number of reported side effects (primary outcome) of the D-MCT during a randomized controlled trial and compare the reported side effects to an active control group (CRT). In the present investigation, we followed the definition of side effects by Linden et al.^7^ and Gerke et al.^19^ as unwanted consequences of correctly applied treatment.

## Methods

### Study design

We analyzed data from a randomized controlled trial (registered as NCT03268434 at clinicaltrials.gov; 31/08/2017) that compared the efficacy of the D-MCT to an active control intervention (CRT: MyBrainTraining). Following baseline assessment (t0), patients were randomized either to the D-MCT or the MyBrainTraining according to a randomization plan (1:1 ratio). Patients received their allocated treatment for 8 weeks (i.e., a maximum of eight sessions) in addition to full access to care as usual (CAU). Patients were reassessed after 8 weeks (t1) and again after 6 months (t2). All patients provided written informed consent. The ethics committee of the German Psychological Association (Deutsche Gesellschaft für Psychologie) approved the study design (LJ05207) and all procedures contributing to this work comply with the ethical standards of the relevant national and institutional committees on human experimentation and with the Helsinki Declaration of 1975, as revised in 2008. Patients received 60€ reimbursement for their expenses if they attended all assessments.

### Patients and procedures

We recruited 84 adult outpatients with unipolar depressive disorder via the outpatient clinic Asklepios Clinic North (Hamburg, Germany) as well as leaflets sent to local psychiatrists and psychotherapists providing outpatient care. Inclusion criteria were a diagnosis of a major depressive disorder (single or recurrent episode) or a persistent depressive disorder (dysthymia) (verified by the 7th version of the Mini International Neuropsychiatric Interview with an additional module on dysthymia^30^). Exclusion criteria were lifetime psychotic symptoms (i.e., hallucinations, mania, or delusions if not mood-congruent and part of depression), intellectual disability (estimated IQ < 70 using a vocabulary test), and dementia. After the intervention (t1), 70 patients (retention rate 83.3%) provided data on negative effects (D-MCT: *n* = 36, MyBrainTraining: *n* = 34). At the 6-months assessment (t2), 74 data sets (retention rate 88.1%) were collected (D-MCT: *n* = 38, MyBrainTraining: *n* = 36).

### Treatment conditions

All patients had full access to CAU. For patients who were already receiving outpatient care from the Asklepios Clinic North (70% of the baseline sample)*,* CAU consisted of an individual treatment plan based on the needs of the patients, for example, short supportive therapeutic/psychiatric contacts and medication.

#### Metacognitive training for depression (D-MCT; intervention)

Psychologists with a master’s degree and postgraduate training in CBT and training in D-MCT conducted the D-MCT group in accordance with the German D-MCT manual^21^. Patients received one session per week (approx. 60 min) over a period of 8 weeks, thus covering all eight modules. Treatment was administered with the help of a slide-based presentation, available for download in English at www.uke.de/depression. The contents of the eight modules are as follows: (1) mental filter, overgeneralization, (2) mood-congruent memory, false memories, (3) should statements, disqualifying the positive, black-and-white thinking, (4) perfectionism, self-worth, (5) magnification and minimization, attributional style, (6) dysfunctional coping strategies (e.g., thought suppression), (7) jumping to conclusions, and (8) identification of emotions, emotional reasoning. For a more in-depth description of the techniques used in the D-MCT, the reader is kindly referred to articles by Jelinek et al.^25,26,29^. The therapists in the study were supervised by one of the authors (LJ).

#### MyBrainTraining (active control)

MyBrainTraining is a CRT that trains users’ mental skills (e.g., concentration, memory) and is available online (www.mybraintraining.com). The tasks are similar to typical computer games (e.g., memorizing objects, logic puzzles). The exercises aim to improve mental functions through targeted practice and can be roughly divided into four categories: calculation, logic, memory, and visual perception. Patients in the control group received a session of MyBrainTraining lasting approximately 60 min once a week over the study period of 8 weeks. The difficulty of each task automatically adapted to the performance of the patients. Patients decided which domains they wanted to train in each session. The sessions were administered by clinical psychologists (with a bachelor’s or a master’s degree in psychology; not the same psychologists who administered the D-MCT), but no further instructions were given apart from general instructions at the beginning and the opportunity to ask questions.

### Measures

#### Psychopathology

The Quick Inventory of Depressive Symptomatology (QIDS)^31,32^ used to assess depressive symptoms in addition to the well-established Hamilton Depression Rating Scale (HDRS-17)^33,34^ because the QIDS offers a more detailed differentiation between depressive symptoms (e.g., gaining vs. losing weight). To quantify self-assessed depressive symptoms, we used the Beck Depression Inventory (BDI-II)^35^.

#### Short inventory of the assessment of negative effects (SIAN) (post)

To assess negative effects of the D-MCT, we used the Short Inventory of the Assessment of Negative Effects (SIAN), which was developed on the basis of the structure of the second part of the Inventory for the Assessment of Negative Effects of Psychotherapy (INEP)^14^. We modified the content of the items, however, for the purposes of the current study. Accordingly, items on increase of symptoms were tailored for depression and assessed in more detail (e.g., depressive thoughts). Moreover, because we were evaluating a group treatment conducted by two therapists, we considered most of the events related to malpractice and unethical behavior unlikely (because therapists and patients can supervise and complement each other, there was a group of patients rather than just one patient, and a third psychologist attended ten sessions to rate treatment adherence and to check for malpractice and/or unethical behavior) and thus omitted all of those items. We did not ask patients to specify the cause of a negative effect and instead included attribution to the study’s intervention in the question (e.g., “I feel more burdened because my depressive thoughts have increased *due to* the D-MCT/MyBrainTraining”). The resulting seven items with a 4-point Likert-type scale (“completely applies” to “does not apply at all”) are displayed in Table 2. To depict frequencies of occurrence, the answer categories “does not apply at all” and “somewhat does not apply” were combined. Internal consistency of the SIAN was good, with Cronbach’s α = 0.823.

#### The negative effects questionnaire (NEQ) (6-month assessment)

The NEQ^36^ is a self-report instrument containing 32 items representing adverse or unwanted events. First, patients rate a specific event, reporting whether it occurred during treatment (yes/no). Second, they indicate how negatively the event affected them on a 5-point Likert scale (0–4). Lastly, patients are asked to attribute the negative effect. In its original form, the question differentiates between negative effects attributed to treatment and those attributed to other circumstances. We slightly modified this to enable attribution to the study intervention or other therapies as well as to other circumstances. The NEQ showed good psychometric properties^36,37^. A six-factor structure for negative effects was found^36^: symptoms (e.g., stress, anxiety), quality (e.g., expectations of treatment remaining unfulfilled), dependency (e.g., dependency on treatment/therapist), stigma (e.g., feeling ashamed in front of others), hopelessness (e.g., experiencing more hopelessness), and failure (e.g., feeling less competent). Due to a technical error, we did not collect data on the first item of the NEQ (“I had more problems with my sleep”), so our version was missing one item and only contained 31 items in total. Our NEQ version still had good internal consistency; Cronbach’s α for the full instrument was α = 0.908 and between 0.589 and 0.890 for the six separate factors. “Symptoms” (although missing 1 item) still showed good internal consistency with Cronbach’s α = 0.877.

### Statistical analysis

We conducted an analysis with all patients providing data for negative effects at t1 or t2, respectively. We first investigated the total number of reported negative effects separately for each of the time points using *t*-tests. We also investigated differences in subjective impairment due to negative effects as measured with the NEQ, again using *t*-tests. If the assumption of equal variances was violated, we used the Welch test, and for the smaller subgroups we used the Mann–Whitney U test. Additionally, we used the chi-square test and the Fisher’s exact test (2 × 2 cross table with side effects yes/no vs. MyBrainTraining/D-MCT) to compare the groups in terms of the number of patients who reported at least one side effect. For the NEQ, we also ran a mixed model ANOVA for the number of negative effects differentiated by cause of the adverse event (study intervention/other therapy/other reasons) as the dependent variable and intervention group as a between-subject factor. To further explore nonsignificant differences between the intervention groups, we ran additional calculations (to compare the mean number of side effects as well as subscales related to the study interventions) even if there was no significant interaction effect. For effects sizes, Cohen’s *d*s (with *d *≈ 0.2, *d *≈ 0.5, and *d *≈ 0.8, corresponding to small, medium, and large effects) and η_p_^2^ (with η_p_^2^ ≈ 0.01, η_p_^2^ ≈ 0.06, and η_p_^2^ ≈ 0.14, corresponding to small, medium, and large effects) were calculated.

## Results

### Sample characteristics at baseline (t0)

Table 1 displays the sociodemographic characteristics of the patients at baseline. Group comparisons between the patients who completed and did not complete the t1 or t2 assessment revealed no significant differences in baseline demographic or clinical characteristics (*p* > 0.05).

**Table 1:** Demographics: frequencies, means, and standard deviations (in brackets).

Variable	D-MCT (*n* = 42)	MyBrainTraining (*n* = 42)	Total (*N* = 84)	Statistics
**Sociodemographic variables**				
Age (years)	48.52 (10.86)	45.09 (14.67)	46.81 (12.94)	*t*(75.5) = 1.128; *p* = .227
Gender				*X*^*2*^(1) = 1.197, *p* = .274
Female	20 (48%)	25 (59%)	45 (54%)	
Male	22 (52%)	17 (41%)	39 (46%)	
Years of formal education	11.05 (1.62)	10.95 (1.71)	11.00 (1.66)	*t*(82) = 0.262; *p* = .794
Marital status				*X*^*2*^(2) = 0.069, *p* = .966
Single	19 (45%)	20 (48%)	39 (47%)	
Partner or married	12 (29%)	11 (26%)	23 (27%)	
Separated, divorced, or widowed	11 (26%)	11 (26%)	22 (26%)	
Job status				*X*^*2*^(2) = 0.348, *p* = .840
Working	10 (24%)	12 (29%)	22 (26%)	
Sick leave	13 (31%)	11 (26%)	24 (29%)	
Unemployed or retired	19 (45%)	19 (45%)	38 (45%)	
Alcohol consumption per week (in g)	46.18 (103.65)	29.93 (77.61)	38.06 (91.37)	*t*(82) = 0.813; *p* = .418
Number of MDEs	9.44 (17.56)	7.73 (12.29)	8.59 (15.09)	*t*(80) = 0.510, *p* = .612
Illness duration (in years)	15.36 (11.39)	15.63 (13.69)	15.49 (12.53)	*t*(82) = 0.096, *p* = .924
Medication				*X*^*2*^(4) = 5.986, *p* = .200
Antidepressive	17 (41%)	23 (55%)	40 (48%)	
Neuroleptic	1 (2%)	0 (0%)	1 (1%)	
Combination	13 (31%)	7 (17%)	20 (24%)	
None	6 (14%)	10 (24%)	16 (19%)	
Other	5 (12%)	2 (4%)	7 (8%)	
Hospitalizations	2.07 (1.86)	1.95 (2.09)	2.01 (1.97)	*t*(81) = 0.277, *p* = .782
Number of outpatient psychotherapies	2.19 (1.32)	2.29 (1.25)	2.24 (1.28)	*t*(81) = 0.361, *p* = .719
**Psychopathology**				
BDI-II	29.89 (9.25)	29.31 (8.07)	29.60 (8.64)	*t*(81) = 0.305, *p* = .761
HDRS	13.88 (5.61)	15.09 (5.74)	14.49 (5.68)	*t*(82) = 0.980, *p* = .330
QIDS	11.9 (4.64)	12.26 (3.77)	12.08 (4.21)	*t*(82) = 0.387, *p* = .700
**Current treatment**				
Sessions attended	5.24 (2.43)	5.60 (2.41)	–-	*t*(82) = 0.677, *p* = .501

MDE = major depressive episode, BDI-II = Beck Depression Inventory, HDRS = Hamilton Depression Rating Scale, QIDS = Quick Inventory of Depressive Symptomatology. Medication “other” is, for example, L-Thyroxin, St. John’s wort.

### Discontinuation

Most of the patients provided data at t1 and t2, but not all patients completed the intervention. To illustrate possible negative events, not revealed by the questionnaires, we will provide a brief description of given reasons for discontinuing the study intervention. Of note, some patients attended irregularly (i.e., did not attend all sessions) due to illness or other important appointments, these are not counted as “discontinued”. Three patients in the D-MCT group did not attend any D-MCT session after being randomized: One could not be reached after baseline assessment and therefore did not provide a reason, the other two indicated being sick at one session (common cold or stomach flu) and later conveyed that the treatment did not fit with their calendar. Two patients in the D-MCT group discontinued the intervention after one and four sessions respectively because they deemed the study “too stressful”. Seven patients of the MyBrain group discontinued treatment: three because of time issues regarding their work or college education after 1 to 4 sessions, one reported the study as “too stressful” after attending one session, one had a recurrence of cancer and for this reason did not wish to continue after two sessions, one did not enjoy using computers (obligatory for MyBrain) and stopped attending after three sessions, and lastly, one found the treatment “ineffective” after two sessions.

Following our definition of the introduction for dropout as an unwanted event, two patients in the D-MCT group (who found the study “too stressful”) and two patients in the MyBrain group (one who found the study “too stressful”, and one who found the treatment “ineffective”) can be considered an unwanted event (e.g., “dissatisfied” dropout^9^).

### Negative effects during and after the Intervention (8-week assessment, t1)

Patients receiving D-MCT (*M* = 0.86, *SD* = 1.17) did not differ significantly from patients who received MyBrainTraining (*M* = 1.00, *SD* = 1.54) regarding the average number of negative effects reported in the SIAN, *t*(68) = 0.426, *p* = 0.671, *d* = 0.103. Eighteen (50%) patients from the D-MCT group and 20 patients (58.8%) from the MyBrainTraining group reported at least one negative effect. The number of patients who reported negative effects for each item in the SIAN is shown in Table 2. The groups did not statistically differ in the number of negative effects.

**Table 2:** Number of patients reporting negative effects in the SIAN (relative ratio in brackets).

Item	D-MCT *(n* = 36)	MyBrain-Training (*n* = 34)	Statistics
1. I am disappointed that I did not feel any better at the end of D-MCT/MyBrainTraining	11 (30.6%)	16 (47.1%)	*X*^*2*^(1) = 2.010, *p* = .156, *d* = 0.344
2. I feel more burdened because my depressive thoughts have gotten worse due to D-MCT/MyBrainTraining	4 (11.1%)	4 (14.3%)	*X*^*2*^(1) = 0.041, *p* = .839; Fisher’s: *p* = 1.000, *d* = 0.048
3. Due to participation in D-MCT/MyBrainTraining, I have fallen into a crisis	4 (11.1%)	4 (11.8%)	*X*^*2*^(1) = 0.007, *p* = .932; Fisher’s: *p* = 1.000, *d* = 0.02
4. Due to D-MCT/MyBrainTraining, my problems have more weight in my life; I am thinking about my problems more than before	5 (13.9%)	4 (11.8%)	*X*^*2*^(1) = 0.070, *p* = .791; Fisher’s: *p* = 1.000, *d* = 0.063
5. I feel like I have changed since my participation in D-MCT/MyBrainTraining and no longer like myself	1 (2.8%)	2 (5.9%)	*X*^*2*^(1) = 0.411, *p* = .522; Fisher’s: *p* = .609, *d* = 0.154
6. The D-MCT/MyBrainTraining sessions burdened me so that I now I have new problems (e.g., poor sleep, decreased appetite)	4 (11.1%)	2 (5.9%)	*X*^*2*^(1) = 0.610, *p* = .435; Fisher’s: *p* = .674, *d* = 0.188
7. I was almost always afraid to join the D-MCT group/MyBrainTraining	2 (5.6%)	2 (5.9%)	*X*^*2*^(1) = 0.003, *p* = .953; Fisher’s: *p* = 1.000, *d* = 0.013

The reported chi-square test was based on a 2 × 2 cross table (side effects yes/no vs. MyBrainTraining/D-MCT).

### NEQ (6-month assessment, t2)

Over all 31 items of the NEQ, the two groups (MyBrainTraining and D-MCT) did not differ regarding the average number of negative effects, *t*(72) = 0.683, *p* = 0.497, *d* = 0.159. A mixed model ANOVA for number of negative effects with cause of adverse event (study intervention/other therapy/other reasons) as a within-subject factor and intervention group as a between-subject factor revealed no significant main effects for cause of negative effects, *F*(2, 144) = 2.223, *p* = 0.112, η_p_^2^ = 0.030 or for intervention group, *F*(1, 72) = 0.402, *p* = 0.528, η_p_^2^ = 0.006. The intervention group x cause of negative effects interaction was also nonsignificant, *F*(2,144) = 0.648, *p* = 0.524, η_p_^2^ = 0.009. See Table 3 for negative effects broken down by subjective cause.

**Table 3:** Mean number of negative effects broken down by cause (standard deviations in brackets).

Cause	D-MCT (*n* = 38)	MyBrainTraining (*n* = 36)	Total (*N* = 74)
Study intervention	2.42 (4.96)	1.81 (3.82)	2.12 (4.42)
Other therapy	1.29 (2.56)	2.00 (3.68)	1.64 (3.15)
Other circumstances	2.68 (4.15)	3.50 (4.65)	3.08 (4.39)
Total	7.21 (6.85)	8.31 (6.93)	7.74 (6.86)

#### Side effects associated with study intervention

Side effects (i.e., negative effects attributed to the study intervention) did not differ between patients of the D-MCT (*M* = 2.42, *SD* = 4.96) and of MyBrainTraining (*M* = 1.80, *SD* = 3.82), *t*(72) = 0.596, *p* = 0.553, *d* = 0.14.

Over all 31 items, 19 patients (52%) reported at least one side effect related to MyBrainTraining, whereas 13 patients (34%) reported at least one side effect related to the D-MCT. No statistically significant association between reporting at least one side effect and intervention group was found, *X*^*2*^(1) = 2.597, *p* = 0.107, however, with a Cohen’s *d* of 0.381, effects were small. See Table 4 for side effects broken down by category. The subjectively rated impairment due to side effects associated with the study intervention did not differ between the D-MCT (*M* = 1.83, *SD* = 0.77) and MyBrainTraining (*M* = 1.95, *SD* = 0.83), *t*(30) = 0.423, *p* = 0.675, *d* = 0.15.

**Table 4:** Number of patients who reported at least one side effect associated with the study intervention.

Factor	D-MCT (*n* = 38)	MyBrainTraining (*n* = 36)
Symptoms*	10 (26.3%)	8 (22.2%)
Quality	9 (23.6%)	15 (41.7%)
Dependency	1 (2.6%)	0 (0%)
Stigma	1 (2.5%)	3 (8.3%)
Hopelessness	5 (13.2%)	2 (5.6%)
Failure	4 (10.5%)	4 (11.1%)

Multiple answers were possible.

*The subscale “symptoms” of the Negative Effects Questionnaire (NEQ) was missing one item.

The mean number of side effects and subjectively rated impairment associated with the study treatment are presented, broken down by intervention group, in Table 5.

**Table 5:** Mean number of side effects (SE) and rated impairment rates (standard deviations in brackets) associated with the study intervention.

Factor	D-MCT (*n* = 38)		MyBrainTraining (*n* = 36)		Statistics	
	Number of SE (SD)	Impairment (SD)	Number of SE (SD)	Impairment (SD)	Number of SE	Impairment
Symptoms*	0.97 (2.03)	2.09 (0.97)	0.56 (1.54)	1.59 (0.72)	*t*(72) = 0.993, *p* = .334	*t(*16) = 1.177, *p* = .256
Quality	0.89 (2.26)	1.83 (1.05)	0.86 (1.38)	2.06 (0.92)	*t*(72) = 0.077, *p* = .939	*t(*22) = 0.575, *p* = .571
Dependency	0.03 (0.16)	n.a.^a^	0 (0)	n.a	*t*(37) = 1.00, *p* = .324	n.a.^a^
Stigma	0.03 (0.16)	3 (0)^b^	0.08 (0.28)	1.67 (0.58)	*t*(55.45) = 1.063, *p* = .292	*U*(*n*_*1*_ = 1, *n*_*2*_ = 3) = 3.009, *p* = .500
Hopelessness	0.26 (0.76)	2 (0.72)	0.14 (0.68)	2.5 (0.71)	*t*(72) = 0.739, *p* = .463	*U*(*n*_*1*_ = 4, *n*_*2*_ = 2) = 2.000, *p* = .533
Failure	0.23 (0.75)	1.63 (0.95)	0.17 (0.56)	2.5 (1)	*t*(72) = 0.454, *p* = .652	*U*(*n*_*1*_ = 4, *n*_*2*_ = 4) = 4.500, *p* = .343

If the assumption of equal variances was violated, the Welch’s t-test was used. For the smaller subgroups, the Mann–Whitney U test was used (*n*_1_ = D-MCT, *n*_2_ = MyBrainTraining). n.a. = not available.

*The subscale “symptoms” of the Negative Effects Questionnaire (NEQ) was missing one item.

^a^Only 1 patient rated dependency, but they did not provide a score for impairment.

^b^Only 1 patient rated stigma.

## Discussion

The aim of the study was to identify side effects of the D-MCT during a randomized controlled trial and compare the reported side effects to an active control group (CRT, MyBrainTraining). After the intervention (post), 50% of the D-MCT patients and 58.8% of the MyBrainTraining patients reported at least one side effect. We classify the reported negative effects as measured with the SIAN as side effects of the respective interventions according to Linden et al.^7^ and Gerke et al.^19^, assuming that no malpractice or unethical conduct occurred. To ensure correctly applied treatment, the therapists delivering the D-MCT received supervision and manual adherence was rated repeatedly during the study. The most frequently reported side effect was disappointment in the subjective benefit of the study treatment. After follow-up, 52% of the MyBrainTraining and 34% of the D-MCT patients reported at least one side effect associated with the study intervention. Of note, the reported ratios for the two time points are not equivalent as we used different questionnaires to measure side effects. At follow-up assessment, the most frequently reported side effects for both interventions fell into the categories of “symptoms” (e.g., stress, anxiety) followed by “quality” (e.g., patients’ expectations of treatment remaining unfulfilled). These results also fit our dropout events, where three patients found the study “too stressful” and one did not see subjective benefit.

Studies have shown that client expectations regarding therapy can influence therapeutic alliance, level of session positivity, and treatment outcome^38–40^ and that patients and therapists have different views of what works in psychotherapy^41^. It is therefore important to induce hope at the beginning of therapy to utilize the positive effect of expectations on therapy outcome. As mid-treatment expectations also relate to treatment outcome^39^, it is also important to regularly check patients’ expectations and to respond accordingly to reduce unrealistic expectations or reinforce past successes^38,42^. In order to manage patients’ expectations and to prevent disappointment, we suggest educating patients comprehensively before treatment. This education should address possible treatment outcomes (e.g., D-MCT can help reduce cognitive biases but does not produce “miracles”—i.e., patients will not necessarily reach full remission), targets of therapy (e.g., a CRT might improve cognitive abilities but not necessarily mood), potential side effects (e.g., being confronted with cognitive biases or cognitive deficits might negatively impact self-esteem), and additional therapy options (e.g., adjunctive individual therapy, medication). This may be done by providing written leaflets in addition to verbal information offered by the therapist. Then, patients can make an informed choice regarding their personal targets for therapy (e.g., if patients desire improvement in cognitive impairments, they probably will choose CRT).

We would also like to discuss two possible reasons for the most reported side effect, which was disappointment in the subjective benefit of the study treatment One reason for the subjective lack of benefit in this investigation may have to do with the specific sample. As Table 1 shows, illness duration, number of hospitalizations, and especially number of depressive episodes were all high in our sample. One could consider the sample as “chronic”^43,44^. Individuals who experience a long illness duration and a high number of treatments and are still regularly seeing a therapist/psychiatrist in an outpatient facility likely represent a rather treatment-resistant population. Therefore, they may have had low expectations before enrolling in this study or, in contrast, had unreasonably high expectations (“This is my last chance”’). Both attitudes could have influenced how the patients reflected on the subjective benefit of treatment (e.g., “Another failed attempt”). This fits the current knowledge of possible risk factors for side effects: The number of pretreatments^18^ or prior experience with psychotherapy^19^ and less optimism about the treatment^17^ or, in contrast, greater optimism about psychotherapy^19^, have already been mentioned in the literature.

Another reason may be due to the different targets of the interventions. As patients could not choose which intervention they received, it is possible that they did not get their desired intervention and therefore did not benefit regarding their own subjective problems. For example, cognitive impairment can differ a lot between depressive patients and over the course of illness^45^. Those with subjectively severe cognitive impairments may have preferred to receive the CRT because they are aware of their impairment, while someone with small or subjectively non-existent cognitive impairments would not expect a benefit (and quite possibly cannot benefit) from the CRT.

Regarding dropout, we agree with O’Keefee et al.^9^ that reasons for dropout should be taken into account, as it is difficult to classify what counts as a dropout in form of an unwanted event. However, we did not extensively interview patients about their reasons for dropouts, and it is therefore difficult to differentiate between different categories—for example between “too stressful” and “time constraints”. The former reason fitting more the category of developing additional symptoms due to therapy, while the latter could also fit the definition of the “dissatisfied” type of O’Keefee et al.^9^, assuming patients were too polite to say so. Consequently, our dropout descriptions should be taken with a grain of salt and future studies on side effects and should try to systematically survey patients’ reasons for dropout.

Complementary to the high acceptance rates found in previous studies of the D-MCT^29^, at post assessment the majority of patients (94.4%) did not report being distressed right before attending the D-MCT sessions and most patients (89%) denied experiencing a crisis or additional symptoms during the intervention period. Also, the majority of D-MCT patients denied stigmatization (97%), which supports the open, unprejudiced atmosphere promoted by the D-MCT. Lastly, as expected from a group therapy setting, the vast majority did not report any adverse effects regarding dependency. At all time points and over all negative effects asked about in the questionnaires, the D-MCT was comparable in number of negative effects to the cognitive remediation training (MyBrainTraining). None of the patients in the MyBrainTraining group developed a dependency on therapy, as is expected with CRTs, which provide little contact with therapists. The most frequently mentioned side effect among MyBrainTraining participants in this study was lack of subjective benefit, which is especially important in combination with the potential detrimental effect of the lack of subjective improvement during a training on self-esteem found in one study^20^. Therefore, for both treatment options, managing expectations seems to be crucial to prevent potential negative side effects.

Although it is difficult to directly compare reported side effects among studies, we want to compare our results with populations that are similar in regard to mental disorder (depression), therapeutic setting (outpatient care) and intervention (D-MCT shows some overlap with CBT): Our results are comparable to one study on negative effects reported by depressive patients in which about half (52.6%) of the patient sample experienced at least one adverse event, of which 38.5% were side effects^15^. Regarding outpatient care our results are in the middle of two published studies: our results are higher compared to the results of Gerke et al.^19^ (15.2%), but considerably lower compared to another outpatient study^18^ in which 84% reported at least one negative effect. Additionally, our follow-up results fit a study on side effects of CBT outpatients in which negative effects in the category “symptoms” were also most frequently reported^18^. It should be noted that the variability in reported side effects probably also stems from the different instruments used to measure negative effects in the studies mentioned. Considering that every treatment comes with potential side effects, both treatments in the current study had relatively tolerable negative effects.

### Limitations

We adapted the second part of the INEP to assess negative effects at post assessment in the current study and used a different questionnaire (NEQ) at the follow-up assessment. Thus, percentages are not comparable between the assessment points. At the same time, this might also be considered a strength as using the two questionnaires allowed us to ask for a wider range of possible side effects. The NEQ version we used was missing one item due to a technical error, but nonetheless the internal consistency remained good and the results should therefore still be interpretable. Of note, the newly developed SIAN has not been validated yet. Future studies should therefore aim to do so and to corroborate our results by additional investigations. We consider malpractice and unethical behavior less likely in a group setting, because two therapists can supervise and complement each other, and because a third psychologist visited ten sessions to rate treatment adherence. While we think systematic malpractice and unethical behavior may have been captured by the adherence ratings, we cannot fully rule them out in the unrated sessions or between sessions. The distinction of negative events that arise in correct therapies from malpractice or unethical behavior seems especially important to safeguard patients’ wellbeing and should therefore be studies in future trials. Of note, therapists’ qualifications differed between the two interventions as the MyBrainTraining was administered by clinical psychologists (B.Sc. or M.Sc.) without postgraduate training in psychotherapy. As MyBrainTraining was designed to be used without any therapeutic guidance at all, we do not think the different therapists’ qualifications between interventions affects study quality. This study was the first to investigate side effects of the D-MCT and therefore we consider it exploratory in nature. Thus, we followed the suggestion of Bender and Lange^46^ and did not correct for multiple testing. Although correction for multiple testing would have reduced the probability of false positive findings, it would also have increased the type II error rate. It thus might have led to missing potentially relevant associations in this emerging field of research, which explains our exploratory strategy. Future studies on side effect of the D-MCT should correct for multiple testing. Of note, our exploratory study might have been somewhat underpowered, but when looking at the effect sizes there was no evidence for large differences between groups. Lastly, a possible allegiance effect should be considered as the authors of the MCT and D-MCT were involved in this study.

### Conclusions

As a follow up to previous studies showing high acceptance rates^29^ as well as good efficacy^25,26^, the number of negative effects was investigated for the first time. The results suggest that both D-MCT and MyBrainTraining are safe and well-received treatment options for people with depression. However, patients’ expectations should be managed to prevent disappointment in the therapy. Future studies on the D-MCT and MyBrainTraining should also measure negative effects and monitor reasons for dropout to corroborate our results.
